# Amyloid Peptide Induced Neuroinflammation Increases the P2X7 Receptor Expression in Microglial Cells, Impacting on Its Functionality

**DOI:** 10.3389/fncel.2019.00143

**Published:** 2019-04-12

**Authors:** Carlos Martínez-Frailes, Caterina Di Lauro, Carolina Bianchi, Laura de Diego-García, Álvaro Sebastián-Serrano, Lisardo Boscá, Miguel Díaz-Hernández

**Affiliations:** ^1^Department of Biochemistry and Molecular Biology, Veterinary School, Complutense University of Madrid, Madrid, Spain; ^2^Instituto de Investigación Sanitaria del Hospital Clínico San Carlos, Madrid, Spain; ^3^Instituto de Investigaciones Biomedicas “Alberto Sols”, Consejo Superior de Investigaciones Científicas-Universidad Autónoma de Madrid, Madrid, Spain

**Keywords:** senile plaques, neuroinflammation, LPS, BzATP, ATP, Alzheimer disease, phagocytosis, migration

## Abstract

Alzheimer disease is a neurodegenerative disease characterized by the presence of senile plaques composed of amyloid-β (Aβ) peptide, neurofibrillary tangles, neuronal loss and neuroinflammation. Previous works have revealed that extracellular ATP, through its selective receptor P2X7 (P2X7R), is essential to neuroinflammation and neurotoxicity induced by Aβ. P2X7R is upregulated on microglial cells around the senile plaques. This upregulation progressively rises with age and is parallel with an accumulation of senile plaques and also correlates with the synaptic toxicity detected both in animal models reproducing AD and human patients of AD. Furthermore, the late onset of the first AD-associated symptoms suggests that aging associated-changes may be relevant to the disease progression. Thus, microglia motility and its capacity to respond to exogenous ATP stimulus decrease with aging. To evaluate whether the P2X7R age related-changes on microglia cells may be relevant to the AD progression, we generated a new transgenic mouse model crossing an Aβ peptide mouse model, J20 mice and the P2X7R reporter mice ^P2X7R^EGFP. Our results indicate that neuroinflammation induced by Aβ peptide causes changes in the P2X7R distribution pattern, increasing it s expression in microglial cells at advanced and late stages, when microgliosis occurs, but not in the early stages, in the absence of microgliosis. In addition, we found that P2X7R activation promotes microglial cells migration to senile plaques but decreases their phagocytic capacity. Moreover, we found a significant reduction of P2X7R transcription on neuronal cells at the early and advanced stages, but not at the late stages. Since previous studies have reported that either pharmacological inhibition or selective downregulation of P2X7R significantly improve behavioral alterations and reduce the incidence and size of senile plaques in the early and advanced stages of AD, the results presented here provide new evidence, indicating that this therapeutic approach could be also efficient in the late stages of the disease.

## Introduction

Alzheimer disease (AD) is a neurodegenerative disease for which, currently, there is no effective treatment available. At a neuropathological level, the disease is characterized by the presence of a profound cortical atrophy associated with a generalized neuroinflammation state (Gahtan and Overmier, 1999), synaptic contacts loss, neuronal depletion and two histopathological hallmarks, the extracellular amyloid plaques and the intracellular neurofibrillary tangles (Selkoe, 2001; Avila, 2006). Neurofibrillary tangles are assembled by hyperphosphorylated tau protein whereas the amyloid plaques are primarily composed of Aβ-amyloid (Aβ) peptide, which is derived from sequential proteolysis of the APP by β- and γ-secretases (Selkoe, 2001). In 2–5% of AD cases have been found mutations both in APP protein and enzymes related in its processing (PS1 and PS2). These cases are denominated as Familiar Alzheimer disease or FAD (Price and Sisodia, 1998; Ling et al., 2003). The development of different transgenic animal models that mimic the FAD symptoms have allowed the confirmation that Aβ peptide is one of the toxic species involved in AD (Hsiao et al., 1996; Mucke et al., 2000; Radde et al., 2006).

Over recent years, several pieces of evidence, provided by different groups, indicate that extracellular ATP plays a crucial role in APP-induced toxicity, primarily through the activation of its selective ionotropic receptor P2X7 (P2X7R). It was found that ATP regulates APP processing via P2X7R (Delarasse et al., 2011; Leon-Otegui et al., 2011; Diaz-Hernandez et al., 2012). A negative association between the P2X7R 489C > T polymorphism and AD has also been established (Sanz et al., 2014). Upregulation of P2X7R was found in AD patients (McLarnon et al., 2006; Sanz et al., 2009; Martin et al., 2018), especially in microglial cells around the senile plaques (Parvathenani et al., 2003). Interestingly, P2X7R upregulation was seen to progressively rise with age and parallels the accumulation of senile plaques (Lee et al., 2011), and correlates with the synaptic toxicity associated to AD (Lee et al., 2011). In agreement with these findings, i.c. of Aβ peptides induced gliosis, hippocampal neurons loss and an increase of hippocampal P2X7R expression (Ryu and Mclarnon, 2008), and the pharmacological P2X7R-blockage reverted the memory impairment associated (Chen et al., 2014). The fact that i.c. of Aβ to P2X7R deficient mice did not cause the above alterations mentioned, suggests that P2X7R is essential for the microglial activation induced by Aβ peptide (Sanz et al., 2009). Furthermore, it also reported that its selective downregulation promotes microglial phagocytosis of Aβ peptide (Ni et al., 2013). Nevertheless, the late onset of the first AD-associated symptoms suggests that aging associated-changes may be relevant to the disease progression (Mosher and Wyss-Coray, 2014). In accordance with this hypothesis, age induced-changes on microglial morphology are exacerbated in AD patients (Serrano-Pozo et al., 2013). Microglia motility and its capacity to respond to exogenous ATP stimulus decrease with aging (Koenigsknecht-Talboo et al., 2008; Damani et al., 2011). It has consequently been seen that microglial cells are removing Aβ in early stages more efficiently than in later stages of AD (Solito and Sastre, 2012). All these data suggest that P2X7R age related-changes on microglia cells are relevant to the AD progression.

In previous studies, we found that *in vivo* pharmacological P2X7R blockage reduced the number and size of senile plaques downregulating the amyloidogenic processing and promoting the non- amyloidogenic processing of APP in young J20 mice, a FAD mouse model (Diaz-Hernandez et al., 2012). However, J20 mice treated with P2X7R antagonist did not show, either a decreased microglial recruitment toward senile plaques or a significant increase in microglial population, at least, at the tested-age (Diaz-Hernandez et al., 2012). Taking into account the repercussion that the microglia aging appears to have on AD progression, in the current study, we decided to analyze whether P2X7R-regulated microglial functions, such as microglial activation, phagocytosis or migration are altered over the AD progression. To address this question, we generated a new transgenic mouse by crossing the AD mouse model, J20 mice, and the P2X7R reporter mice ^P2X7R^EGFP.

## Materials and Methods

### Animals

All animal procedures were carried out at the Universidad Complutense of Madrid, in compliance with National and European regulations (RD1201/2005; 86/609/CEE) following the guidelines of the International Council for the Laboratory Animal Science. The protocol was approved by the Committee of Animal Experiments of the Complutense University of Madrid and the Environmental Counseling of the Comunidad de Madrid, Spain. All animals were housed with food and water available *ad libitum* and maintained in a temperature-controlled environment on a 12/12 h light/dark cycle with light onset at 08:00 A.M. All surgery was performed under isoflurane anesthesia, and all efforts were made to minimize suffering.

^P2X7R^EGFP reporter mice (Tg [P2rx7-EGFP] FY174Gsat/Mmcd, stock 011959-UCD) expressing EGFP immediately downstream of P2X7R promoter (Sebastian-Serrano et al., 2016). J20 hAPP transgenic mouse line express a mutant form of the human amyloid protein precursor bearing both the Swedish (K670N/M671L) and the Indiana (V717F) mutations (APPSwInd), labeled as strain B6.Cg-Tg (PDGFB-APPSwInd) 20Lms/2J. This mouse strain develops the characteristic amyloid peptide deposits by 6–8 months of age (Mucke et al., 2000). ^P2X7^EGFP/J20 mice were generated crossing heterozygous ^P2X7^EGFP mice by heterozygous J20 mice.

### PCR Genotyping

Genomic DNA was obtained from tail biopsies using Wizard^®^ SV Genomic DNA Purification System (Promega, Madison, WI, United States) according to the manufacturer’s protocol.

Simple PCR reactions were carried out using DNA Amplitools Master Mix (Biotools, Madrid, Spain), specific primers (400 nM each) and 5 μL of genomic DNA in a final volume of 25 μL. Animals were genotyped using specific primers for ^P2X7R^EGFP Fw 5′-CCTACGGCGTGCAGTGCTTCAGC-3′ and Rv 5′-CGGCGAGCTGCACGCTGCGTCCTC-3′; primers for J20 Fw 5′-GGTGAGTTTGTAAGTGATGCC-3′ and Rv 5′-TCTTCTTCTTCCACCTCAGC-3′. PCR was carried out over 40 cycles of 94°C for 30 s, 60°C for 45 s, and 72°C for 45 s for EGFP primers or over 40 cycles of 94°C for 30 s, 60°C for 45 s, and 72°C for 45 s for J20 primers.

PCR amplification products were electrophoresed on a 1.5% (w/v) agarose gel and stained with SYBR^®^ Safe DNA Gel Stain (Life Technologies, Carlsbad, CA, United States). PCR bands were visualized by gel imaging system Gel Logic 200 Imaging System (Kodak, Rochester, NY, United States).

### Human Samples

The Netherlands Brain Bank provided the human brain tissues, which supplies postmortem specimens from clinically well documented and neuropathologically confirmed AD patients and non-diseased donors (NBB, Netherlands Institute for Neuroscience, Amsterdam). The NBB works following all national laws and regulations. Frozen samples used were obtained from three different regions of the temporal lobe (inferior, medial, and superior) from four patients with the clinical diagnosis of AD (three women aged 73, 83, and 85 years old and one man aged 85 years) and four non-demented controls (three women aged 70, 72, and 85 years old and one man aged 73 years) following the protocols of nervous tissue donation approved by the local Ethical Committees of the Netherlands Brain Bank. The postmortem delay in tissue processing was between 4 and 5 h in both groups.

### Microglial Cell Culture

Primary microglial cultures were prepared from the hippocampus of WT at postnatal (p) 4–5 days as previously described (de Diego Garcia et al., 2018). Mixed glial culture containing both astrocytes and microglial was maintained in a 37°C incubator with a humidified atmosphere of 5% CO_2_ for 14 days in 75 cm^2^ flasks containing DMEM medium supplemented with 10% fetal calf serum (Gibco) 2 mM glutamine, 100 μ/ml penicillin, 100 μg/ml streptomycin, 50 μg/ml kanamycin, and 0.25 μg/ml Amphotericin B (all from Sigma-Aldrich). The medium was replaced every 3 days. Microglial cells were de-attached by shaking the culture flasks at 250 rpm for 2 h at 37°C in an orbital shaker. Microglia cells were plated at 5 × 10^5^ cells/well in six-well plates and cultured in supplemented DMEM media (Thermo Fisher).

### Chemical and Antibodies

LPS, ATP, BzATP (catalog number B6396), and the antibody anti-α-tubulin (catalog number T6199) were purchased from Sigma-Aldrich (Madrid, Spain). Anti-NeuN antibody (catalog number MAB377) were obtained from Merck Millipore (Tullagreen, Ireland). A438079 (catalog number 2972/10) was obtained from Tocris BioScience (Bristol, United Kingdom). Monoclonal anti Aβ 4–10 residues clone WO2 (catalog number MABN10) was purchased from Merck Chemicals (Madrid, Spain). The commercial antibody for P2X7R (catalog number APR-004) was purchased from Alomone Labs (Jerusalem, Israel). Antibodies against enhanced green fluorescent protein (EGFP) were obtained from Thermo Fisher (catalog number A11122) and AVESLab (catalog number GFP-1020) (Tigard, OR, United States). Red Fluorescent 2 μm diameter microspheres (catalog number F8826) (Life technologies). Antibody against Iba-1 (catalog number 019-19741) was provided from Wako (Richmond, VA, United States). CytoD (catalog number sc-201442) was provided by Santa Cruz (Dallas, TX, United States). Selective P2X7R antagonist GSK 1482160A was provided by GlaxoSmithKline (Madrid, Spain)

### Phagocytosis Assays

*In vitro*; Phagocytosis assays were performed 24 h after microglial cell seeding on coverslips of 35-mm of diameter. Pharmacological treatments were applied 20 min before adding the fluorescencent microspheres. Red Fluorescent 2 μm diameter microspheres (Life Technologies) at 0,002% in DMEM medium were added to microglial cells, and afterward, cells were put back in the incubator and left to rest for 2 h. Then, the medium was removed, and cells were washed thoroughly with PBS three times and fixed in PFA 4%.

Microglial cells were stained with a rabbit polyclonal anti-Iba1 (1/200) obtained from Wako and revealed with goat anti-rabbit IgG labeled with Alexa 488 (1/400) obtained from Molecular Probes. For each coverslip, 4 random pictures were taken, using the TCS SPE confocal microscope (Leica Microsystems, Wetzlar, Germany). Images were analyzed using ImageJ software (US National Institutes of Health, Bethesda, MD, United States). The number of fluorescent beads inside each cell was counted, and the average number of phagocyted microspheres per microglial cell was calculated for each experiment and treatment. Experiments were repeated at least 3 times for each condition.

In vivo; 6-month-old ^P2X7R^EGFP mice (*n* = 6) were anesthetized with isoflurane (1-chloro-2, 2, 2-trifuoroethyl-difluormethylether) (Isovet^®^, BRAUN, Rubi, Barcelona, Spain) diluted in 50% O_2_. The scalp was incised along the midline, and one hole was made at the appropriate stereotaxic coordinates from Bregma (mediolateral, 1 mm; anteroposterior, 2 mm; dorsoventral, 1.8 mm). 1 μL of 0.02% red Fluorescent 2 μm microspheres in PBS was intracranial administrated (i.c.) at a rate of ≈1 μL/min.

In some cases, the selective P2X7R antagonist GSK 1482160A (from GlaxoSmithKline, Madrid, Spain) was i.p. administrated at 100mg/Kg dosage every 24 h for 1 week. GSK 1482160A was diluted in vehicle solution composed of 20% hydroxypropyl-β-cyclodextrin plus 0.2% DMSO in sterile PBS. Control littermates were treated with vehicle solution using the same relationship (volume per body weight).

### Migration Assay

The migration assays were performed placing 1.5 × 10^5^ hippocampal microglia cells contained in 200 μL of serum free in the upper chamber of the 24-well format, 8.0 μm pore size *trans*-well inserts (Costar, New York, NY, United States), and 600 μL of the same medium containing 10% of FBS in the lower chamber. In some cases, cells were pre-treated with 1 μM GSK 1482160A before stimulating them with 1 mM ATP or 300 μM BzATP for 24 h at 37°C in 5% of CO_2_. The inserts were subsequently removed rinsing twice with PBS and fixed in 4% PFA for 10 min, followed by staining with DAPI for 10 min. The cells on the upper side of the filter were removed with cotton-tipped swabs. The microglial migration was quantified by counting the number of cells that migrated through the membrane to the other side. The images were selected from 20 random fields for each treatment with microscopy at 50x magnification, and the number of migrating cells was quantified. Each experiment was repeated at least three times.

### LPS Treatment

^P2X7R^EGFP mice weighing at the indicated ages were i.p. treated with either sterile PBS or LPS (5 mg/Kg, *Escherichia coli*, serotype 055: B5) (*n* = 5 per age and treatment). Mice were sacrificed 24 h (acute) or 7 days (chronic) after injection.

### Immunofluorescence Studies

For confocal microscopy, animals were transcardially perfused with 4% PFA in Sorensen’s buffer for 10 min, post-fixed, and brains were cryoprotected in sucrose before sectioning. Brain slices were washed in PBS and treated with blocking solution containing 5 % FBS, 1% BSA, and 0.2% Triton-X 100 in PBS buffer. After that, samples were incubated with primary antibodies diluted in blocking solution. After washed them, sections were incubated with fluorescent-tagged secondary antibodies to be counterstained with DAPI (Thermo Fisher) and mounted in FluorSave (Merck Millipore) later. The following primary antibodies were used at the indicated dilutions: mouse anti-APP clone (WO2) 1:200, rabbit anti-GFP 1:400, chicken anti-EGFP 1:400, rabbit anti-P2X7R 1:200, rabbit anti-Iba-1 1:200. Donkey anti-rabbit, anti-mouse or anti-chicken secondary antibodies, conjugated with Alexa 488, 594 or 647 (Life Technologies, Madrid, Spain) were used at 1:400. Confocal images were acquired with a TCS SPE. Confocal images stacks were acquired with a TCS SPE microscope from Leica Microsystems equipped with a Plan Fluor 10X dry objective lens NA = 0.30, 40X Apochromat NA = 1.15 oil objective lens and 63X Apochromat NA = 1.3 oil objective lens (Leica Microsystems GmbH) at room temperature. The images stacks are composite by a sequence of at least 20 pictures acquired every 0.5 μm on the *Z*-axis. For the detection of DAPI, 405 nm laser line was used. For Alexa Fluor 594, the 561 nm laser line was used. For Alexa Fluor 488, the 488 nm laser line was used. For Alexa 647, the 645 nm laser line was used. Images were acquired using the Leica software LAS AF v2.2.1 software (Leica Microsystems GmbH) and the Z projection of images stacks was made using ImageJ 1.47d (NIH). In some cases, it also shows orthogonal views corresponding to specific locations (indicated by dashed lines) of images stack. Green cells in the hippocampus were considered those cells positively marked with antibodies against GFP with an identifiable cellular morphology and whose nucleus was positively stained with DAPI.

### Quantification of GFP Positive Microglial and Neuron Cells and Distances Measurement

For each case (at least 4 mice per phenotype, age and treatment) 4 series (16 sections, 90 μm apart, approximately spanning from Bregma −0.95 mm to −3.78 mm according to Paxinos and Franklin (2001) of sections (30 μm thick) were selected to be immunostained with antibodies anti-GFP, anti-Iba-1, anti-P2X7R or anti-APP (clone WO2) respectively. In some cases, hippocampal slices were stained with more than one antibody.

The number of cells corresponding to each cell type was counted, identified by specific antibodies (neurons using NeuN antibody and microglial cells with Iba-1 antibody). The counting was performed using blind-coded on grayscale 8-byte images from whole-hippocampal area acquired at low magnification. The background signal was determined by visual analysis, obtaining a cut-off value of 150 on a 0–255 scale with 0 = white and 255 = black using the free software ImageJ 1.45h (US National Institutes of Health, Bethesda, MD, United States).

Distances of GFP positive or P2X7R positive cells to the senile plate were measured from the core of the cellular body, to the border of the nearest senile plate bigger than 30 nm in its longer axis.

### Western Blotting

Hippocampal samples from WT, J20, ^P2X7R^EGFP or ^P2X7R^EGFP/J20 mice and Human samples were treated with lysis buffer containing 20 mM HEPES, 100 mM NaCl, 50 mM NaF, 5 mM EDTA, 5 mM Na_3_VO_4_ (all salts from Sigma-Aldrich), 1% Triton X-100, okadaic acid and CompleteTM Protease Inhibitor Cocktail Tablets, pH 7.4 (Roche Diagnostics GmbH). Protein concentration was determined, and then, samples were boiled in a gel-loading buffer and separated by SDS-PAGE. Proteins were transferred to nitrocellulose membranes and probed with the following primary antibodies: rabbit anti- P2X7R (1:1000) antibody or mouse anti-α-tubulin (1:10000) antibody. Blots were then washed in PBS-T and incubated with goat anti-rabbit or goat anti-mouse IgGs coupled with HRP (Amersham GE Healthcare), used at 1:1000 and 1:5000, respectively. Protein bands were visualized by chemiluminescence (Pierce Biotechnology, Rockford, IL, United States) using ImageQuant LAS500 (GE Healthcare Life Sciences) and analyzed using ImageJ software (v1, 47d, NIH, Bethesda, MD, United States).

### Statistical Analysis

Results were analyzed by unpaired *t*-test or ANOVA test followed by Bonferroni’s or Sidak’s multiple comparisons tests using GRAPH PAD PRISM 6 (Graph Pad Software Inc., San Diego, CA, United States) and expressed as the s.e.m. Differences were considered to be significant at *p* < 0.05.

## Results

### In a Familiar Alzheimer Disease Mouse Model (FAD), Microglial Cells Begin to Express P2X7R at Adult Stages, Once Microgliosis Takes Place

**FIGURE 1:**
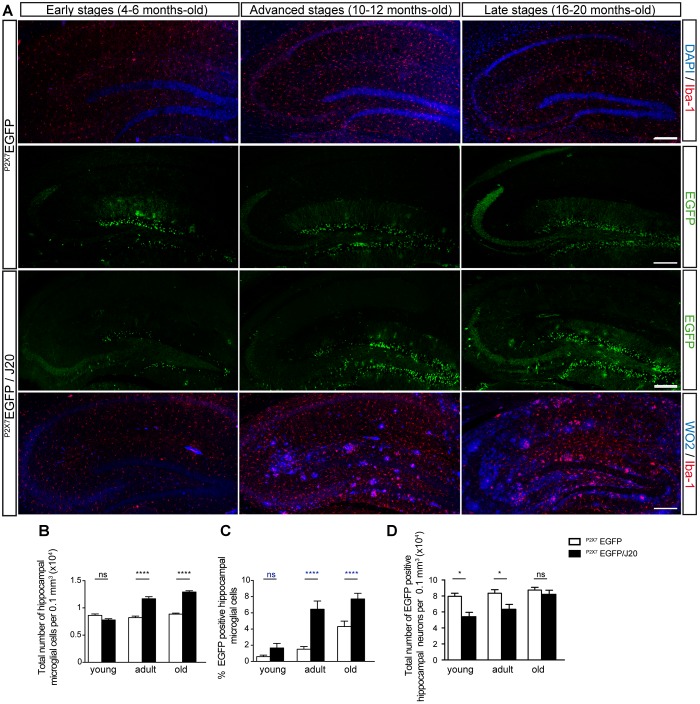
Microgliosis in ^P2X7R^EGFP/J20 mice promotes increased expression of the P2X7R reporter protein in microglial cells and reduces it in neurons. **(A)** Representative images of hippocampal coronal sections from ^P2X7R^EGFP and ^P2X7R^EGFP/J20 mice at 4–6 months-old (early stage), 10–12 months-old (advanced stage) and 16–20 months-old (late stage). Sections from ^P2X7R^EGFP mice stained with nuclear marker DAPI plus microglial marker Iba-1 or antibodies against EGFP. Sections from ^P2X7R^EGFP/J20 mice were stained with antibodies against EGFP or antibodies against APP protein (WO2) plus antibodies against Iba-1. Scale bar: 200 μm. **(B)** The total number of microglia cells per 0.1 mm^3^ of the hippocampal section on ^P2X7R^EGFP mice (white bars) and ^P2X7R^EGFP/J20 mice (black bars) at each analyzed age. **(C)** Percentage of hippocampal EGFP positive microglial cells on ^P2X7R^EGFP mice (white bars) and ^P2X7R^EGFP/J20 mice (black bars) at each indicated age (*n* ≥ 5 mice per genotype and age; sections ≥ 9 per mouse). **(D)** Quantification of EGFP positive neurons per 0.1 mm^3^ of the hippocampal section on ^P2X7R^EGFP mice (white bars) and P2X7REGFP/J20 mice (black bars) at each analyzed age (*n* ≥ 5 mice per genotype and age; sections ≥ 9 per mouse). ^∗^*p* < 0.05 and ^∗∗∗∗^*p* < 0.0001 using an unpaired *t*-test, ns not statistically significant. Data in bar graphs represent mean ± s.e.m.

To elucidate if Aβ peptide conditions the P2X7R expression on microglial cells along the AD progression, we generated a new transgenic mouse model by crossbreeding the amyloid mice model of the AD, J20 mice with the ^P2X7R^EGFP mice that express EGFP under the control of the P2X7R promoter. ^P2X7R^EGFP/J20 and their corresponding control littermates ^P2X7R^EGFP mice were sacrificed to analyze their hippocampus by immunofluorescence techniques at the following ages; 4–6 months-old (early stage), 10–12 months-old (advanced stage), and 16–20 months-old (late stage). Results showed that EGFP positive cells were mainly found on the GLDG on both genotypes and at all ages tested. In addition, disseminated EGFP-positive cells throughout the whole hippocampus were also found (Figure 1A). Double immunofluorescence studies using specific neuronal marker NeuN or glial markers GFAP and Iba-1 for astroglial cells and microglial cells respectively revealed that EGFP positive cells on GLDG were neurons while disseminated extra granular EGFP-positive green cells were microglial cells (Supplementary Figure 1). Curiously, in the early and advanced stages, the number of EGFP-positive neurons in GLDG was lower in ^P2X7R^EGFP/J20 than observed in ^P2X7R^EGFP mice (7.9 ± 0.4 10^4^ vs. 5.4 ± 0.5 10^4^ in the early stages and 8.3 ± 0.4 10^4^ vs. 6.3 ± 0.6 10^4^ in the advanced stages, Figure 1A,D). However, no change on EGFP-positive neurons in GLDG was found between ^P2X7R^EGFP/J20 and ^P2X7R^EGFP mice at late stages (8.7 ± 0.3 10^4^ vs. 8.2 ± 0.5 10^4^ in the late stages, Figure 1A,D). Otherwise, ^P2X7R^EGFP/J20 mice presented a higher number of disseminated EGFP-positive microglial cells than those observed in their corresponding control littermates ^P2X7R^EGFP mice both in the advanced and late stages (1.5 ± 0.3 vs. 6.4 ± 1.0 in the advanced stages and 4.3 ± 0.6 vs. 7.7 ± 0.6 in late stages, Figure 1A,C). Non-differences in the number of EGFP-positive microglial cells were found between ^P2X7R^EGFP/J20 and ^P2X7R^EGFP mice in the early stages (0.6 ± 0.2 vs. 1.6 ± 0.5, Figure 1A,C). It is worth highlighting that while ^P2X7R^EGFP/J20 and ^P2X7R^EGFP mice showed, in the early stages, a similar number of hippocampal microglial cells, in the advanced and late stages, ^P2X7R^EGFP/J20 mice presented a significant increase of microglial cells compared with those detected in their corresponding littermates ^P2X7R^EGFP mice (Figure 1A,B). These data indicate that in the FAD early stages, when a microgliosis had not yet taken place but the senile plaques start to be detected (Figure 1A), negative modulation of P2X7R transcription occurs in neurons, but did not affect microglial cells. Conversely, in FAD advanced stages, once a significant microgliosis begins to be detected and the number of senile plates increase (Figure 1A,B), a rise of P2X7R transcription occurs in microglial cells, but the reduction of P2X7R transcription is maintained in neurons. Finally, in the late stages of the FAD, when microgliosis remains over time, the increased of P2X7R transcription remains in microglial cells, but the neuronal reduction of P2X7R transcription disappears.

**FIGURE 2:**
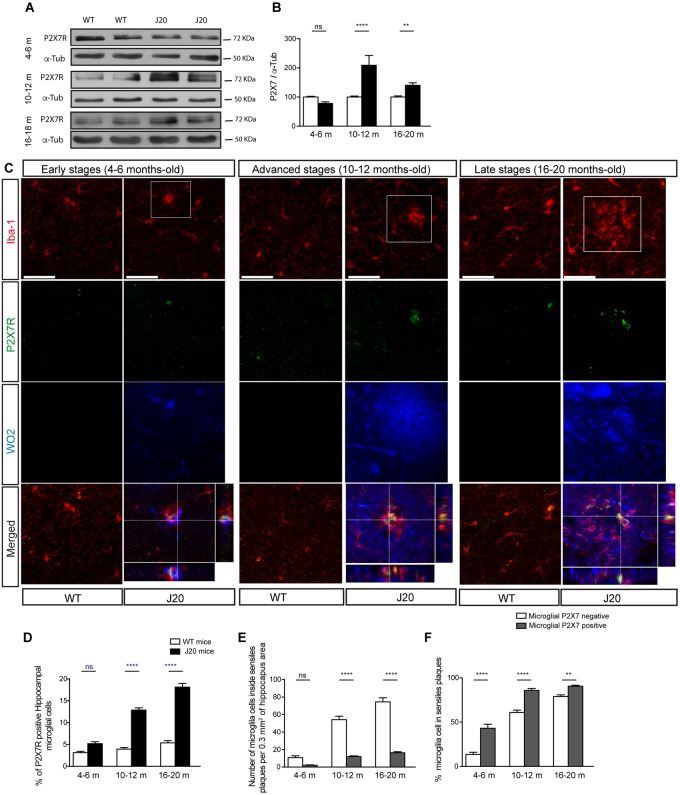
J20 mice increase the P2X7R expression levels in microglial cells at advanced and late stages when they develop a significant microgliosis. **(A)** Representative images of Western blot using hippocampal samples from J20 at 4–6 months-old (early stage), 10–12 months-old (advanced stage), and 16–20 months-old (late stage) and their corresponding control-littermates (WT) mice stained with antibodies against constitutive P2X7R or α-tubulin. **(B)** Graphs show quantification of the protein expression of P2X7 receptor (*n* ≥ 6 mice per genotype and age). Data were normalized using α-tubulin levels. The 100% value indicates the corresponding P2X7R protein expression detected in WT mice. ^∗∗^*p* < 0.01 and ^∗∗∗∗^*p* < 0.0001 using unpaired *t*-test. **(C)** Representative micrographs of hippocampal sections from J20 and WT mice at early, advanced or late stages stained with antibodies against P2X7 receptor (green), microglial marker Iba-1 (red), and APP protein (WO2) (blue). Merged images both the full field and a magnification 1.5x of the area indicated in upper images are shown at the bottom with their corresponding orthogonal views. Dashed lines represent the locations where orthogonal views were obtained. Scale bar: 50 μm. **(D)** The graph shows the percentage of positive P2X7R microglial cells in WT (white) or J20 (black) mice per 0.1 mm^3^ of hippocampal section (*n* ≥ 5 mice per genotype and age; sections ≥ 10 per mouse). **(E)** Quantification of the total number of microglial cells expressing (gray) or not P2X7R (white) inside of senile plaques per 0.3 mm^3^ of the hippocampal section. **(F)** Percentage of hippocampal microglial cells expressing (gray) or not P2X7R (white) inside of senile plaques (*n* ≥ 5 mice per genotype and age; sections ≥ 8 per mouse). The 100% value corresponded to the total number of microglial cells expressing or not P2X7R. ^∗∗^*p* < 0.01 and ^∗∗∗∗^*p* < 0.0001 using an unpaired *t*-test, ns not statistically significant. Data in bar graphs represent mean ± s.e.m.

In the following step, we decided to check if the changes observed in P2X7R transcription affected the protein expression of this receptor. To this end, we measured P2X7R protein expression levels in hippocampal samples from WT and J20 mice at the same ages as those we had previously analyzed ^P2X7R^EGFP/J20 and ^P2X7R^EGFP mice. Results revealed that J20 mice, at the early stages, presented similar P2X7R levels to those observed in their control littermates. However, in their advanced and late stages, J20 mice showed higher P2X7R expression levels than those detected in their corresponding control littermates (209 ± 34% in the advanced stages and 140 ± 9% in the late stages, Figure 2A,B). Double immunofluorescence studies using P2X7R and Iba-1 antibodies showed that, similarly to that observed in ^P2X7R^EGFP/J20 mice, J20 mice presented a higher number of P2X7R positive microglial cells than those detected in their corresponding control littermate WT mice both in the advanced and late stages (4 ± 1% vs. 13 ± 1% in the advanced stages and 5 ± 1% vs. 18 ± 1% in the late stages (Figure 2C,D). Interestingly, something similar was observed in ^P2X7R^EGFP mice, the percentage of positive P2X7R microglial cells was low in WT mice at all ages tested (Figure 1C, 2C). When we analyzed the distribution pattern of P2X7R positive microglial cells, we found them mainly localized inside senile plaques at all ages tested (Figure 2F). However, P2X7R positive microglial cells were not abundant at this location (Figure 2E). It is also worth noting that the J20 mice did not show any significant differences, either in the total number (12.0 ± 1.2 and 15.3 ± 1.2 respectively), or in the percentage of EGFP-positive microglial cells (9 ± 2% and 90 ± 2% respectively) inside the senile plates between their advanced and late stages (Figure 2E,F).

**FIGURE 3:**
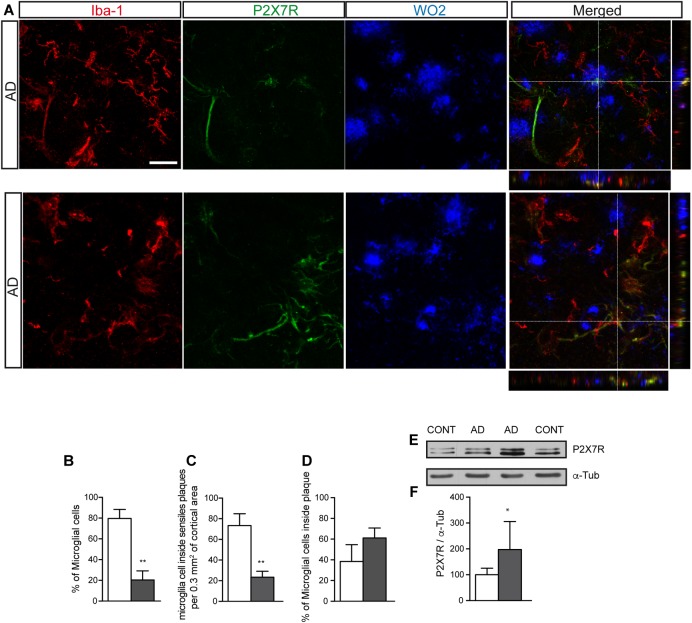
Human AD patients showed a significantly increased P2X7R expression in microglial cells, preferably localized inside of senile plaques. **(A)** Representative images of post-mortem cortical sections from AD patients stained with antibodies against P2X7 receptor (green), microglial marker Iba-1 (red) and APP protein (WO2) (blue). Merged images and orthogonal views are also shown. Dashed lines represent the locations where orthogonal views were obtained. Scale bar: 20 μm. **(B)** The graph shows the percentage of microglial cells expressing (gray) or not (white) P2X7R in post-mortem cortical sections from human AD patients (*n* = 4; sections ≥ 10 per case). **(C)** Quantification of the total number of microglial cells expressing (gray) or not P2X7R (white) inside of senile plaques per 0.3 mm^3^ of the cortical area. **(D)** Percentage of hippocampal microglial cells expressing (gray) or not P2X7R (white) inside of senile plaques. (*n* = 4; sections ≥ 10 per case). The 100% value corresponded to the total number of microglial cells. ^∗∗^*p* < 0.01 using unpaired *t*-test. **(E)** Representative images of Western blot using post-mortem cortical samples from human AD patients or non-demented controls stained with antibodies against P2X7R or α-tubulin. **(F)** Graphs show a quantification of the protein expression of P2X7 receptor (*n* = 4 AD and *n* = 4 controls). Data were normalized to the expression levels of α-tubulin. The 100% value indicates the corresponding P2X7R protein levels detected in non-demented controls. ^∗^*p* < 0.05 using unpaired *t*-test. Data in bar graphs represent mean ± s.e.m.

Similar to what was observed in J20 mice at advanced and late stages, we detected a significantly increased of P2X7R expression in samples from human AD patients (Figure 3E,F). Immunofluorescence studies using human brain samples confirmed that P2X7R is expressed in microglial cells (Figure 3A), although the number of microglial cells expressing P2X7R was lower than those non-expressing this receptor (Figure 3B). Regarding their distribution pattern, we observed, once again, that positive P2X7R microglial cells were preferentially inside extracellular β-amyloid deposits (Figure 3C,D). These results confirm that J20 mice mimics the microglial P2X7R location observed in human AD patients.

### Neuroinflammation Changes the P2X7R Distribution Pattern

Once we had observed that, both in FAD mouse models and human AD patients, the increased P2X7R expression occurred in microglial cells, we decided to identify the factors causing it, as well as its consequences on microglial functionality.

**FIGURE 4:**
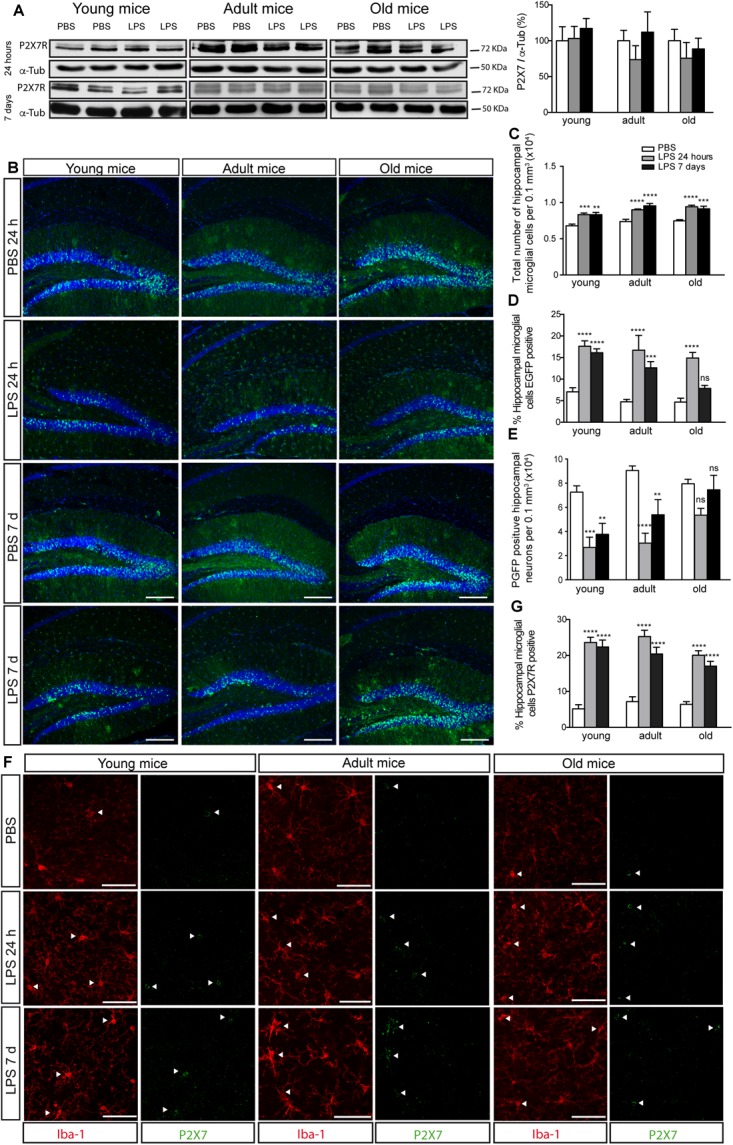
LPS-induced neuroinflammation increases P2X7R in microglial cells and reduces its levels in neurons. **(A)** Representative images of Western blot using hippocampal samples from young (4–6 months–old), adult (10–12 months old), or old ^P2X7R^EGFP mice (16–20 months-old) intraperitoneally PBS- or LPS-treated for 24 h or 7 days and stained with antibodies against P2X7R or α-tubulin. The graph shows the quantification of P2X7R protein levels (*n* ≥ 5 mice per age and treatment). Data were normalized using α-tubulin levels. The 100% value corresponds to P2X7R protein levels detected in PBS-treated mice. Data in bar graphs depict mean ± s.e.m. **(B)** Representative images of hippocampal coronal sections from young, adult or old ^P2X7R^EGFP mice intraperitoneally PBS- or LPS-treated for 24 h or 7 days and stained with an antibody against EGFP plus nuclear marker DAPI (lower panels). Scale bar: 200 μm. **(C)** Quantification of the total number of microglial cells per 0.3 mm^3^ of the hippocampal section on young, adult or old ^P2X7R^EGFP mice intraperitoneally PBS- or LPS-treated for 24 h or 7 days. **(D)** Percentage of hippocampal EGFP positive microglial cells on young, adult or old ^P2X7R^EGFP mice intraperitoneally PBS- or LPS-treated for 24 h or 7 days. 100% value corresponded to the total number of microglial cells expressing EGFP in PBS-treated mice. **(E)** Graphs show the total number of hippocampal neurons expressing EGFP on young, adult or old ^P2X7R^EGFP mice intraperitoneally PBS- or LPS-treated for 24 h or 7 days (*n* ≥ 5 mice per age and treatment; sections ≥ 8 per mouse). ^∗∗^*p* < 0.01, ^∗∗∗^*p* < 0.001, and ^∗∗∗∗^*p* < 0.0001 using ANOVA test followed by Bonferroni’s tests, ns not statistically significant. Data in bar graphs depict mean ± s.e.m. **(F)** Representative micrographs of hippocampal sections from young, adult or old ^P2X7R^EGFP mice intraperitoneally PBS- or LPS-treated for 24 h or 7 days and stained with antibodies against P2X7 receptor (green) and microglial marker Iba-1 (red). Arrowheads show the microglial cells expressing P2X7R. Scale bar: 50 μm. **(G)** The graph shows the percentage of hippocampal microglial cells expressing P2X7R on young, adult or old ^P2X7R^EGFP mice intraperitoneally PBS- or LPS-treated for 24 h or 7 days (*n* ≥ 5 mice per age and treatment; sections ≥ 10 per mouse). ^∗∗∗∗^*p* < 0.0001 using ANOVA test followed by Bonferroni’s tests. Data in bar graphs represent mean ± s.e.m.

As, in studies using J20 mice, we observed that the P2X7R distribution pattern changed when a significant increase in microglial cells took place, initially, we decided to evaluate if the associated neuroinflammation process might be one of the factors involved in it. To assess this hypothesis, we decided to resort to the LPS-induced neuroinflammation model. Additional groups of young, adult and old ^P2X7R^EGFP mice were treated i.p. with LPS or vehicle solution for 24 h or 7 days. Results showed that, although the LPS administration did not modify the P2X7R hippocampal expression levels (Figure 4A), it did, however, induce a similar P2X7R distribution pattern change to the one observed in ^P2X7R^EGFP/J20 mice (Figure 4B). LPS-treated ^P2X7R^EGFP mice presented a significant increase in EGFP-positive microglial cells throughout the hippocampus at all ages tested. This change was independent of the treatment duration (Figure 4B,D). This fact was linked to a significant increase in the total number of hippocampal microglial cells (Figure 4C). Curiously, only 7 days-LPS-treated ^P2X7R^EGFP old mice did not show a significant increase of EGFP-positive microglial cells when compared to their respective 7 days-vehicle-treated ^P2X7R^EGFP old mice (4.6 ± 0.9 vs. 7.8 ± 0.7, Figure 4B,D). Moreover, we found, once more, in young and adult LPS-treated ^P2X7R^EGFP mice, a significant decrease in the EGFP-positive neurons in GLDG (63.1 ± 7.2% and 48.1 ± 10.5% for 24 h or 7 days of LPS-treatment respectively on young mice and 66.4 ± 9.0% and 40.5 ± 12.9% for 24 h or 7 days of LPS-treatment respectively on adult mice, Figure 4B,E). However, this reduction was not observed in old LPS-treated ^P2X7R^EGFP mice independently of the treatment duration (Figure 4B,E). To validate the results obtained with ^P2X7R^EGFP reporter protein, we performed a new analysis using anti-P2X7R antibodies. Results revealed that LPS-treatment induced a significant increase in P2X7R-positive microglial cells at all ages tested (Figure 4F,G). It is important to consider that, by using this approach, we found a significant increase in the number of P2X7R-positive microglial cells even in LPS-treated old mice (Figure 4F,G). These data are suggesting that P2X7R distribution pattern change detected in J20 mice may be caused by the associated neuroinflammation developed by mice during the progression of the disease.

### P2X7R Regulates the Microglial Cell Recruitment to Senile Plate

**FIGURE 5:**
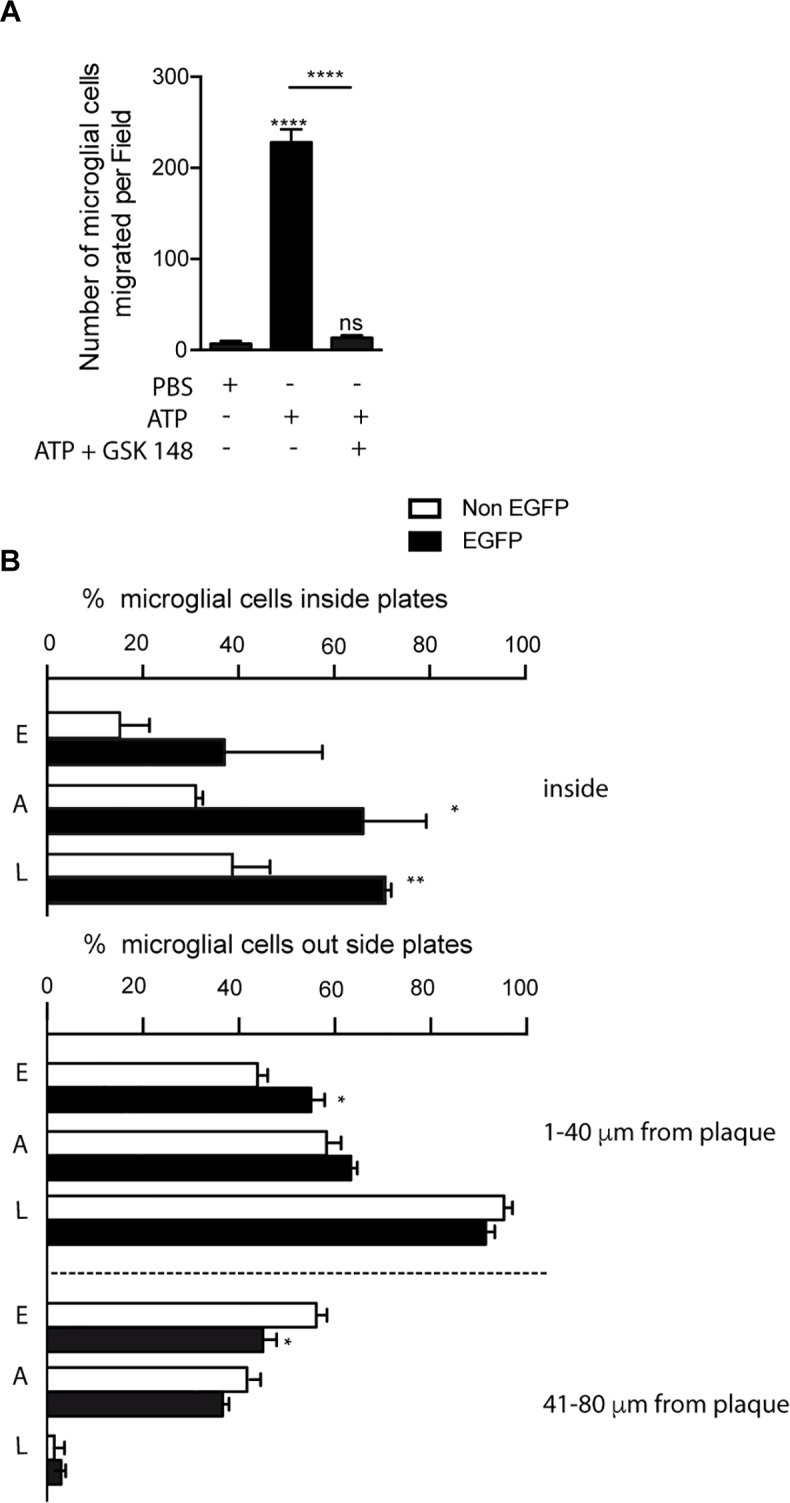
Selective P2X7R activation promotes microglial migration to senile plates. **(A)** The graph shows the number of cultured microglial cells from the hippocampus of WT mice migrating through trans-well inserts after being stimulated with 1 mM ATP in the presence or absence of 1 μM GSK 1482160A. Values represent, at least, the mean ± s.e.m of 3 independent cultures run in duplicate. ^∗∗∗∗^*p* < 0.0001 using ANOVA test followed by Bonferroni’s tests, ns not statistically significant. **(B)** Graphs show the number of hippocampal microglial cells expressing (black bars) or not (white bars) the P2X7 receptor reporter protein (EGFP) inside (upper graph) or in the nearness (lower graph) of senile plaques identified ^P2X7R^EGFP/J20 mice at early, advanced or late stages (*n* ≥ 5 mice per age; sections ≥ 8 per mouse; plaques ≥ 15 per sections). ^∗^*p* < 0.05, ^∗∗^*p* < 0.01, using unpaired *t*-test. Data in bar graphs represent mean ± s.e.m. E meaning early stages, A meaning advances stages and L meaning late stages.

Taking into account that our preliminary data revealed that P2X7R positive microglial cells were mainly inside senile plates, we wondered if P2X7R favors the migration of microglial cells toward them. To address this point, initially, we decided to evaluate the role of P2X7R on microglial cell migration, using primary microglial cells cultures isolated from the hippocampus. Results showed that high ATP concentration (1 mM) promotes microglial cells migration (6.9 ± 3.0 migrated microglial cells stimulated by PBS vs. 228.2 ± 14.2 migrated microglial cells ATP-stimulated, Figure 5A). Since acute stimulation of P2X7R by high ATP concentrations can induce cellular death (Miras-Portugal et al., 2017), we decided to stimulate the microglial cells with lower concentrations of ATP (0.3 mM) or with the selective P2X7R agonist BzATP (0.3 mM, Supplementary Figure 2). Both stimulations caused similar results on microglial mobility than that induced high ATP concentration (Supplementary Figure 2). The involvement of P2X7R was confirmed when the selective P2X7R antagonist GSK 1482160A prevented the microglial migration induced by 1mM ATP (13.5 ± 2.6 migrated microglial cells after being stimulated by ATP in presence GSK 1482160A, Figure 5A) or 300 μM BzATP (Supplementary Figure 2). These results point to the fact that P2X7R is regulating the microglial mobility, and, consequently, ATP may be considered as a chemotaxis signal for microglial cells.

In the following step, to determine whether P2X7R regulates microglial cells recruitment to senile plates, we decided to analyze ^P2X7R^EGFP/J20 mice. In this study, we measured the number of both EGFP-positive and EGFP-negative microglial cells inside senile plates, as well as the distance between the center of microglial cell to the border of the most closed senile plate. As shown in Figure 5B, in all ages tested we observed higher percentages of EGFP-positive than EGFP-negative microglial inside the senile plates, although only in the advanced and late stages this increase was significant (37.1 ± 11.8% vs. 15.2 ± 3.5% respectively in young mice, 66.1 ± 7.6% vs. 31.1 ± 0.8% respectively in adult mice and 71.2 ± 1.1% vs. 38.7 ± 4.5% respectively in old mice, Figure 5B). Furthermore, in the early stages, we detected a higher percentage of EGFP-positive microglial cells at the most closed environment of senile plates than EGFP-negative ones (55.0 ± 2.8% vs. 43.8 ± 2.2% respectively, Figure 5B). On the other hand, we found a lower percentage of EGFP-positive microglial cells than EGFP-negative ones at distances further away from the senile plates (45.0 ± 2.8% vs. 56.1 ± 2.2% respectively, Figure 5B). A similar tendency, but not statistically significative, was found in the advanced stages (63.3 ± 1.3% vs. 58.3 ± 1.0% and 36.6 ± 1.3% vs. 41.6 ± 1.1% respectively, Figure 5B). In the late stages, we did not find any difference in the distribution pattern of microglial cells outside senile plates (91.4 ± 2.0% vs. 95.3 ± 1.7% and 2.9 ± 1.0% vs. 1.5 ± 0.9% respectively). Although our findings suggest that P2X7R-activation promotes the microglial recruitment toward senile plates, in comparison, inside the senile plates, the majority of microglial cells did not express this receptor.

### P2X7R Modules the Phagocytic Capacity of Microglia Cells

**FIGURE 6:**
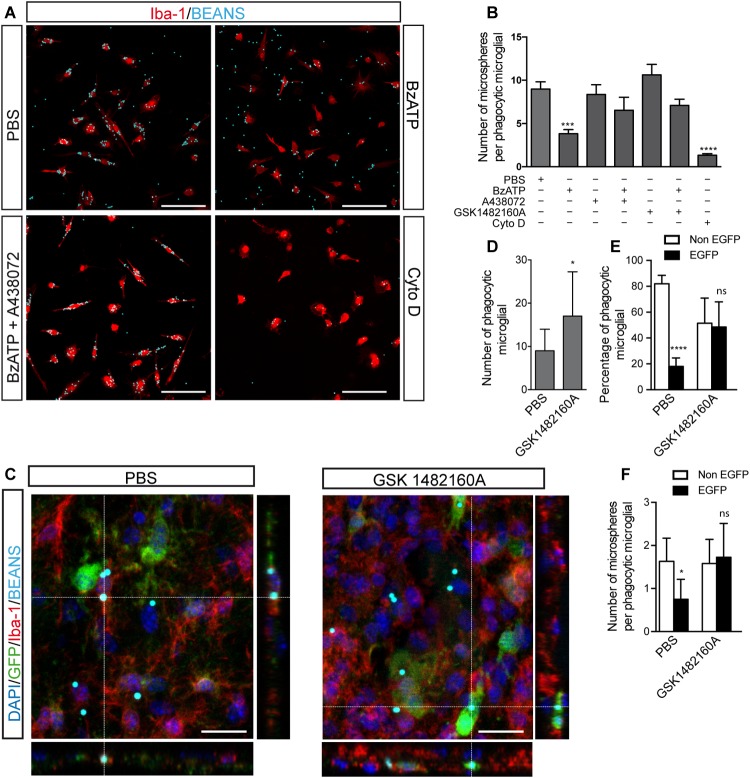
Selective P2X7R inhibition promotes microglial phagocytosis. **(A)** Representative fluorescence micrographs of cultured microglial cells from the hippocampus of WT mice identified using antibodies against the microglial marker Iba-1 (red) uptaking fluorescence microparticles (cyan) after stimulated with 300 μM BzATP in presence or absence of the selective P2X7R antagonists 10 μM A438072 or 1 μM GSK 1482160A. In some cases, the cultured microglial cells were treated with 10 μM cytochalasin D (CytoD) as a negative control. **(B)** The graph shows the number of phagocyted microfluorescent particles per microglial cell under different experimental conditions. Values represent, at least, the mean ± s.e.m of 4 independent cultures run in duplicate. ^∗∗∗^*p* < 0.001 and ^∗∗∗∗^*p* < 0.0001 using ANOVA test followed by Bonferroni’s tests. **(C)** Representative confocal images and its corresponding orthogonal views of hippocampal sections from 6 months-old ^P2X7R^EGFP mice intraperitoneally GSK 1482160A-treated for 7 days after intracranial administration of fluorescent microspheres to the hippocampus. Dashed lines represent the locations where orthogonal views were obtained. Sections were stained with antibodies against EGFP (green), microglial marker Iba-1 (red), nuclear marker DAPI (blue), being microspheres visualized in cyan. Scale bar: 50 μm. **(D)** The graph shows the number of microglial cells uptaking fluorescent microspheres on PBS- or GSK 1482160A-treated ^P2X7R^EGFP mice (*n* ≥ 4 mice per treatment; sections ≥ 8 per mouse). **(E)** The graph shows the percentage of phagocytic microglial cells expressing (black) or not (white) the P2X7 receptor reporter protein EGFP on PBS- or GSK 1482160A-treated ^P2X7R^EGFP mice (*n* ≥ 4 mice per treatment; sections ≥ 8 per mouse). **(F)** The graph shows the number of incorporated fluorescent microspheres by microglial cells expressing (black) or not (white) the P2X7 receptor reporter protein EGFP on PBS- or GSK 1482160A-treated ^P2X7R^EGFP mice (*n* ≥ 4 mice per treatment; sections ≥ 8 per mouse). ^∗^*p* < 0.05, ^∗∗∗∗^*p* < 0.0001, using unpaired *t*-test, ns not statistically significant. Data in bar graphs represent mean ± s.e.m.

To elucidate what role P2X7R plays on recruited microglial cells inside senile plates, we decided to evaluate the possible role of this receptor in another of the most relevant microglial functions, the phagocytosis. Initially, we decided to use, once again, primary microglial cells cultures. To evaluate phagocytic capacity of cultured microglial cells, we measured the number of fluorescence microspheres phagocyted after them were stimulated with BzATP in the presence or absence of the selective P2X7R antagonists (Figure 6A).

Our results showed that BzATP stimulation reduced the phagocytic ability of microglial cells (8.9 ± 0.8 microspheres per microglial cell treated with PBS vs. 3.8 ± 0.4 microspheres per microglial cell treated with BzATP, Figure 6A,B). Interestingly, selective P2X7R antagonists reverted the reduced phagocytic capacity induced by BzATP (6.5 ± 1.5 microspheres per microglial cell treated with A438072 plus BzATP and 7.1 ± 0.7 microspheres per microglial cell treated with GSK 1482160A plus BzATP, Figure 6A,B). As negative phagocytosis control, microglial cells were treated with CytoD, an inhibitor of actin polymerization that blocks more than 90% of the phagocytosis capacity (1.3 ± 0.1 microspheres per microglial cell treated with Cyto D, Figure 6A,B). Interestingly, the negative effect induced by P2X7R activation on microglial phagocytic capacity was not modified when cells were LPS-pre-treated (8.4 ± 0.6 microspheres per microglial cell treated with LPS vs. 2.9 ± 0.8 microspheres per microglial cell treated with LPS plus BzATP, Supplementary Figure 3). To consolidate these results, in the following step, we decided to administrate fluorescence microspheres i.c. to the hippocampus of ^P2X7R^EGFP mice. After i.c. injection, ^P2X7R^EGFP mice were daily i.p. treated for 7 days with the P2X7R antagonist GSK 1482160A or vehicle solution before being sacrificed. Hippocampus from ^P2X7R^EGFP mice was analyzed by immunofluorescence techniques using anti-EGFP, and anti-Iba-1 antibodies (Figure 6C). Results revealed that *in vivo* pharmacology P2X7R blockage promoted the phagocytic capacity of the microglial cells (Figure 6D). Interesting, most of the phagocytic microglial cells on vehicle-treated mice expressed the P2X7R reporter protein EGFP (81.9 ± 2.4% was EGFP-negative vs. 18.1 ± 2.4% was EGFP-positive). However, in GSK 1482160A-treated mice, no significant differences were detected between both microglial populations (51.5 ± 5.0% was EGFP-negative vs. 45.5 ± 5.0% was EGFP-positive) (Figure 6E). It is worth highlighting that P2X7R inhibition also caused the EGFP-positive microglial cells to increase their phagocytic capacity, so these cells passed to phagocyte a mean of 0.7 ± 0.1 to 1.7 ± 0.2 microspheres per cell (Figure 6F). However, the pharmacological blockage of P2X7R did not modify the phagocytic capacity of EGFP-negative cells (1.6 ± 0.2 vehicle vs. 1.5 ± 0.1 GSK 1482160A) (Figure 6F). All these data suggest that P2X7R negatively modulates the microglial phagocyte capacity.

## Discussion

In the present study, we have evaluated the impact of microglial cells expressing P2X7R on FAD progression analyzing the new transgenic mouse model ^P2X7R^EGFP/J20 mice. In the early stages, when Aβ peptide begins assembling on detectable extracellular β-amyloid deposits, but still non-evident microglial proliferation takes place, we did not observe significant changes in the percentage of microglial cells expressing P2X7R. However, at this stage, we found that outside plaques, microglial cells expressing P2X7R were closer to nascent plaques than those non-expressing this receptor. On the other hand, both in advanced and late stages, when J20 mice experience microgliosis and an evident cognitive and motor impairments (Mucke et al., 2000), significant increases in P2X7R expression levels and in the percentage of P2X7R expressing microglial cells were detected. Furthermore, at both stages, although P2X7R-expressing microglial cells were mostly found inside the senile plaques, this microglial population was not the most abundant, either inside the senile plaques or in the whole hippocampus. We also found a significant reduction in the number of neuronal cells transcribing P2X7R in the early and advanced, but not in the late stages.

As observed in J20 mice, we detected in brain samples from AD patients, both a significant increase of P2X7R expression levels and a preferred location of microglial cells expressing P2X7R inside senile plaques, but this was not, once again, the most abundant microglial population at this location. Our data confirm that P2X7R mediated signaling is increased in microglial cells on AD, as previously reported by other groups (Parvathenani et al., 2003; McLarnon et al., 2006; Lee et al., 2011). However, on these works did not identify the factors causing the increased P2X7R expression in microglial cells or what consequences had on microglial functionality.

It is well known that ATP-signaling via P2X7R regulates different microglial functions. It is also reported that ATP-induced morphological changes related with their migratory ability, allowing them rapidly to migrate toward the injury (Davalos et al., 2005), in an ATP-induced ATP-release dependent mechanism (Dou et al., 2012). Our *in vitro* studies confirmed that ATP promotes the microglial mobility by P2X7R activation. Furthermore, using ^P2X7R^EGFP/J20 mice, we have demonstrated that microglial cells expressing P2X7R migrate more quickly toward the extracellular β-amyloid deposits than those non-expressing this receptor. These results suggest that the enrichment of P2X7R-expressing microglial cells around β-amyloid deposits observed in the last stages of AD by other authors (Parvathenani et al., 2003; McLarnon et al., 2006; Lee et al., 2011) may result from the increased migratory capacity of these cells by the increase of P2X7R expression levels. This hypothesis is in accordance with the fact that the upregulation of P2X7R on microglial cells increases parallel to the incidence of senile plaques in the brain (Lee et al., 2011). Moreover, since microglial P2X7R activation also triggers the cytokines release (Di Virgilio et al., 2017), P2X7R activation on migratory microglial would favor, in turn, the recruitment of microglial cells toward the plaques. In this way, we found that outside of extracellular β-amyloid deposits, the non-positive P2X7R microglial cells present delayed recruitment toward them compared to microglial cells expressing P2X7R.

It has also been reported that cytoskeleton changes in microglial cells caused by ATP-induced P2X7R activation, reduce their phagocytic capacity (Fang et al., 2009). Accordingly, downregulation of P2X7R using small RNA interference favors the Aβ peptide phagocytosis (Ni et al., 2013). Here, using both *in vitro* and *in vivo* approaches, we found that selective pharmacological P2X7R inhibition promotes microglial phagocytosis. In agreement with an inverse relationship between P2X7R expression and microglial phagocytic capacity, we discovered that most microglial cells in the senile plaques did not express P2X7R. It is worth highlighting that, although there was a higher number of microglial cells inside senile plaques in the late, rather than in the advanced stages, the percentage of P2X7R positive microglial cells inside extracellular Aβ deposits is similar in both phases. Considering that microglial recruitment toward senile plaques remains over AD progression, one possible explanation is that, after promotes migration of microglial cells to senile plaques, once they reach the plaque, P2X7R expression is decreased favoring the Aβ peptide phagocytosis. Supporting this hypothesis, we found that the low phagocytosis capacity detected in P2X7R-expressing microglial cells increases once the receptor is pharmacologically blocked. However, additional experiments should be made to confirm this hypothesis.

Over recent years, there has been an active debate in the field about the preferential expression of P2X7R on neural cells (Illes et al., 2017; Miras-Portugal et al., 2017). These opposing points of view are mainly due to that fact that some groups found P2X7R in neurons (Miras-Portugal et al., 2017), whereas other groups detected it mainly in glial cells, especially in microglial cells, but not in neurons (Illes et al., 2017). In the present work, using a well-characterized P2X7R reporter mice ^P2X7R^EGFP (Engel et al., 2012; Sebastian-Serrano et al., 2016), we found that, at hippocampal level, under physiological conditions, P2X7R is mainly transcribed in neurons, expressing on the nerve endings from those granular neurons on dentate gyrus that project their axons toward the CA3 region, as previously reported (Sebastian-Serrano et al., 2016). However, under pathological conditions where neuroinflammation causes microglial proliferation and activation, as occurs in J20 mice at advanced and late stages or in LPS treated mice, the P2X7R distribution pattern changes, decreasing its transcription on neurons and increasing in microglial cells. These results suggest that neuroinflammation regulates the expression of P2X7R both in neurons and in glial cells. In agreement with this idea it has been reported that P2X7R pharmacological blockage or its downregulation by small RNA interference attenuated LPS-induced neuroinflammation by avoiding the microglial activation and the subsequence inflammation (Lu et al., 2013), and decreased microglial proliferation (Bianco et al., 2006). Since proinflammatory cytokines released by P2X7R may cause the receptor upregulation, this positive feedback loop might promote and maintain, over time, an exacerbating microglial response contributing in this way to the adverse effects associated with neuroinflammation (Mosher and Wyss-Coray, 2014). Supporting this hypothesis, a close relationship has been detected between P2X7R upregulation in microglial cells and synaptic toxicity on AD (Lee et al., 2011). In this sense, it is worth noting that the compression of brain tissue by the extracellular Aβ deposits may cause a mechanical ATP release (Burnstock et al., 2011), which, in addition to favoring the migration of P2X7R-expressing microglial cells toward these deposits, may also promote a sustained activation of neuronal P2X7R. Since the persistent activation of P2X7R on neurons by high extracellular ATP levels may also lead to compromise the cellular viability, the reduction of P2X7R transcription observed in neurons both the early and advanced stages, may be an adaptive physiological response to avoid or at least reduce the neuronal loss associated to AD. In this way, the loss of this regulatory mechanism might be contributing to the neuronal loss detected in the late stages of AD.

## Conclusion

We found that neuroinflammation associated with AD induces a change in the P2X7R distribution pattern, increasing its expression on microglial cells at advanced and late stages of AD, when there is evident microgliosis. P2X7R activation improves the migratory faculty of microglial cells to senile plaques but decreases their phagocytic capacity. Additionally, we found a significant reduction of P2X7R transcription in neuronal cells at the early and advanced, but not at the late stages of AD. Because different studies have demonstrated that both pharmacological inhibition or the selective downregulation of P2X7R significantly improve behavioral alterations and reduce the incidence and size of senile plaques in the early and advanced stages of AD (Diaz-Hernandez et al., 2012; Chen et al., 2014; Martin et al., 2018), the results presented here provide new pieces of evidences indicating that this therapeutic approach may also be useful in the late stages of the disease.

## Ethics Statement

This study was carried out in accordance with the recommendations of ‘Ethical Committees of the Netherlands Brain Bank with written informed consent from all subjects. All subjects gave written informed consent in accordance with the Declaration of Helsinki. The protocol was approved by the Ethical Committees of the Netherlands Brain Bank.’ This study was carried out in accordance with the recommendations of ‘International Council for the Laboratory Animal Science’. The protocol was approved by the Committee of Animal Experiments of the Complutense University of Madrid and the Environmental Counseling of the Comunidad de Madrid, Spain.

## Author Contributions

CM-F treated and processed the mice generating and analyzing the samples, participated in experimental design, and contributed to the interpretation of the data. CDL contributed the processing and analyzing of samples and contributed to the interpretation of the data. AS-S, LdD-G, CB, and LB revised the manuscript and helped in the interpretation of data. MD-H participated in the experimental design, in the interpretation of the results, wrote the manuscript, and also provided the financial support for the work.

## Conflict of Interest Statement

The authors declare that the research was conducted in the absence of any commercial or financial relationships that could be construed as a potential conflict of interest.

## Abbreviations

Aβ peptide: amyloid-β
AD: Alzheimer disease
APP: amyloid precursor protein
ATP: Adenosine 5′-triphosphate
BSA: bovine serum albumin
BzATP: 2′,3′-O-(4-benzoyl)-benzoyl ATP
CNS: central nervous system
CytoD: Cytochalasin D
DAPI: 4′,6-Diamidine-2′-phenylindole dihydrochloride
DMEM: Dulbecco’s Modified Eagle Medium
DMSO: dimethyl sulfoxide
EGFP: enhance green fluorescent protein
FAD: Familiar Alzheimer disease
FBS: fetal bovine serum
GLDG: granular layer of the dentate gyrus
HEPES: 4-(2-hydroxyethyl)-1-piperazineethanesulfonic acid
HRP: horseradish peroxidase
i.c.: intracranial administration
i.p.: intraperitoneally
J20 mice: mouse model of Familiar Alzheimer disease
p: postnatal day
P2X7R: P2X7 receptor
^P2X7R^EGFP mice: P2X7R reporter mice ^P2X7R^EGFP
^P2X7R^EGFP/J20: mice generated crossing ^P2X7R^EGFP and J20 mice
PBS: Phosphate-Buffered saline
PBS-T: PBS-buffer containing 0.1 % (v/v) Tween-20
PFA: paraformaldehyde
PS1 and PS2: presenilin-1 and -2
s.e.m.: mean ± standard error of the mean
SDS-PAGE: Sodium dodecyl sulfate-polyacrylamide gel electrophoresis
WT: wild-type mice.
