# Supplementary material for: Amyloid Peptide Induced Neuroinflammation Increases the P2X7 Receptor Expression in Microglial Cells, Impacting on Its Functionality
Source: Front Cell Neurosci. 2019 Apr 12;13:143. doi: 10.3389/fncel.2019.00143 (PMC6474397; doi:10.3389/fncel.2019.00143)
Supplement: Supplementary file 1 [file Table_1.DOCX]

Supplementary Material of manuscript entitled “**Amyloid peptide induced neuroinflammation increases P2X7R expression in microglial cells impacting to their functionality**” by Martinez-Frailes et al.


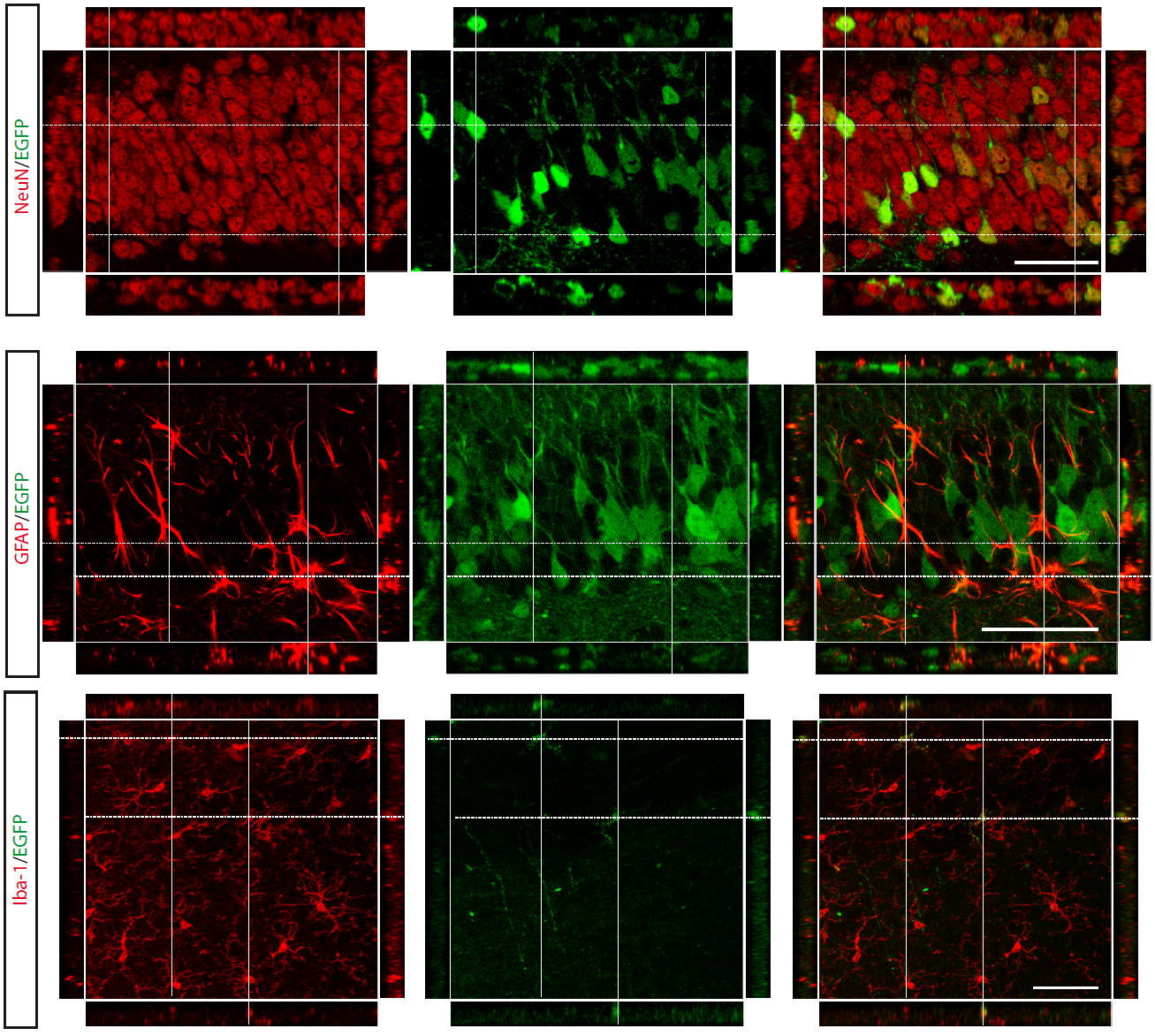


**Supplementary figure 1. Identification of the cellular lineage of EGFP positive cells.** Representative confocal images and orthogonal views of hippocampal sections from ^P2X7R^EGFP adult mice stained with antibodies against neuronal marker NeuN (upper images), astroglial marker GFAP (middle images) or microglial marker Iba-1 (lower images) plus antibodies against EGFP protein. Merged images are also shown. Dashed lines represent the locations where orthogonal views were obtained. Scale bar: 50 µm.


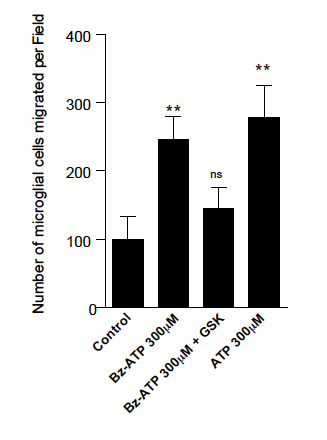


**Supplementary Figure 2. Selective P2X7R activation promotes microglial migration.** The graph shows the percentage of cultured microglial cells from the hippocampus of WT mice migrating through trans-well inserts after stimulated with 300 µM BzATP in the presence or absence of 1 µM GSK 1482160A or with 300 µM ATP. 100% values correspond to the number of microglial cells migrated PBS stimulated. Values represent, at least, the mean ± s.e.m of 3 independent cultures run in duplicate. **p<0.01 using ANOVA test followed by Bonferroni´s tests, ns not statistically significant.

**
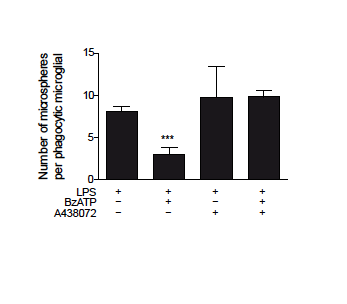
**

**Supplementary Figure 3. LPS-induced neuroinflammation did not affect the negative regulation of microglial phagocytic capacity by P2X7R.** The graph shows the number of up taken microfluorescent particles per microglial cell after stimulated with 50 ng/ml LPS in the presence or absence of with 300 µM BzATP plus selective P2X7R antagonist 10 µM A438072. Values represent, at least, the mean ± s.e.m of 4 independent cultures run in duplicate. ***p<0.001 using ANOVA test followed by Bonferroni´s tests.
